# Ly6d expression delineates two putative postnatal thymus epithelial progenitor cells that are differentially affected by aging

**DOI:** 10.1126/sciadv.aec2253

**Published:** 2026-08-07

**Authors:** Irene Calvo-Asensio, Andreas Tarcevski, Fatima Dhalla, Anja Kusch, Thomas Barthlott, Saulius Zuklys, Georg A. Holländer, Michael D. Morgan

**Affiliations:** ^1^ Department of Biomedicine, University of Basel and University Children’s Hospital, Basel, Switzerland.; ^2^ Institute of Developmental and Regenerative Medicine, University of Oxford, Oxford, UK.; ^3^ Department of Paediatrics, University of Oxford, Oxford, UK.; ^4^ Department of Biosystems Science and Engineering, ETH Zürich, Basel, Switzerland.; ^5^ Botnar Institute of Immune Engineering, Basel, Switzerland.; ^6^ Institute of Medical Sciences, School of Medicine, Medical Sciences and Nutrition, University of Aberdeen, Foresterhill, Aberdeen, UK.

## Abstract

To date, the identity and maintenance of postnatal thymic epithelial progenitor cells (TEPCs) remain unclear, as does the persistence of bipotent TEPCs after birth or whether lineage-restricted progenitors independently maintain separate TEC compartments. Using an inducible lineage-tracing system based on expression of the thymoproteasomal protein β5t, which is expressed in embryonic and a subset of postnatal TEPCs, we explored the early dynamics of the relationships between thymic epithelial cell (TEC) progenitors and their progeny. Our results identified two potential lineage-biased progenitor subpopulations, distinguished by *Ly6d* expression. Additionally, we observed that aging disproportionately affects *Ly6d*^−^ compared to *Ly6d*^+^ TEPCs, with implications for rejuvenation of aging thymic epithelia. This study provides insights into the developmental pathways of TEC lineages and their maintenance, contributing to strategies for enhancing thymic function in aging and disease.

## Teaser

The protein Ly6D identifies epithelial progenitors in the postnatal thymus, pointing to separate maintenance of major lineages.

## INTRODUCTION

The thymus, as a primary lymphoid organ, provides the necessary structural and functional support for the development of naïve T cells collectively expressing an antigen receptor repertoire purged of reactivity to an individual’s tissue antigens yet poised to react to potentially injurious foreign antigens (*1*–*3*). Thymic epithelial cells (TECs), together with mesenchymal, endothelial, and hematopoietic cell types, form the major stromal components. TECs are categorized into separate cortical (c) and medullary (m) lineages on the basis of their specific spatial, molecular, structural, transcriptional and thus functional characteristics. cTECs induce the commitment of blood-borne precursor cells to a T cell fate, foster their subsequent expansion, guide their maturation, and control both positive and negative selection of antigen receptor bearing thymocytes (*4*–*7*). In contrast, mTECs not only promote the terminal differentiation of thymocytes that include the establishment of immunological tolerance to self-antigens via a deletional mechanism (negative thymocyte selection) but also promote the generation of natural regulatory T cells (*8*, *9*). The critical need for T cell antigen receptor–mediated negative selection depends on mTEC’s promiscuous expression of transcripts that encode proteins that are normally only detected in organs other than the thymus. Using flow cytometry, a limited number of markers have been identified that distinguish mTECs from cTECs and that classify separate maturational stages (*10*) such as Ulex europaeus agglutinin 1 (UEA1) lectin reactivity and Ly51, respectively (*11*).

Advances in single-cell RNA sequencing (scRNA-seq) have uncovered a previously unappreciated degree of TEC heterogeneity (*12*–*17*). For clarity, we distinguish flow cytometry phenotypes from scRNA-seq cell types as subpopulations and subtypes, respectively. Using scRNA-seq, we recently described intertypical TEC (*12*), so named because they share transcriptomic properties of both cTECs and mTECs. Intertypical TECs are characterized by the expression of genes previously associated with thymic epithelial progenitor cells (TEPCs) localized at the cortico-medullary junction (CMJ), such as *Pdpn* (*15*, *18*), *Ccl21a* (*18*, *19*), and *Ly6a* (which encodes Sca1) (*20*, *21*), as well as low cell-surface protein levels of expression of major histocompatibility complex (MHC) class II (*22*–*24*).

The thymus is the first organ to display several hallmarks of aging, a process referred to as thymic involution characterized by a loss of cellularity, changes in gross anatomy, and diminished T cell output with an altered antigen receptor repertoire (*12*, *25*). Similarly, external and iatrogenic insults such as cytoablative treatments and infections lead to compromised thymic functions (*23*), resulting in weakened T cell responses to pathogens, diminished immune surveillance, and an elevated susceptibility to autoimmunity and cancer (*26*, *27*). Consequently, over the past few decades, considerable research has been directed toward uncovering strategies that can either preserve or restore thymic functionality (*28*). Research to identify, characterize, and test the mechanisms that support and maintain thymic function by TEPCs suggests the presence of both bipotent progenitors of cTECs and mTECs and corresponding unipotent progenitors for each lineage (*18*–*22*, *24*, *29*–*37*).

During embryogenesis, TECs arise from a common TEPC in the third pharyngeal pouch. Evidence suggests that these embryonic progenitors are bipotent, giving rise to both the cTEC and mTEC lineages. Embryonic TEPCs phenotypically resemble cTECs due to the common expression of canonical cTEC-specific proteins including CD205, β5t, and interleukin-7 (*38*–*40*). In contrast, there is a lack of consensus on the identity of postnatal TEPC and how they maintain the TEC compartment throughout life (*19*, *21*, *22*, *29*, *30*, *34*, *41*). Some reports have suggested that self-renewing bipotent TEPCs persist after birth (*21*, *34*, *37*), whereas others advocate for a series of lineage-restricted progenitors that independently maintain the different TEC compartments during adulthood (*14*, *30*–*33*). In addition, how the newly described TEC subtypes, such as intertypical TEC, perinatal cTEC, or tuft-like mTEC, fit into these different developmental models requires clarification (*38*).

Lineage tracing studies have shown that β5t^+^ cTEC-like TEPCs located at the CMJ can serve as efficient progenitors for mTECs, although their contribution to the mTEC lineage becomes restricted in the postnatal thymus (*18*). This raises the question of whether alternative TEPCs contribute to mTEC maintenance in the adult thymus and what the developmental relationship is between adult and embryonic progenitor populations. We recently suggested that intertypical TEC may constitute a previous missing link between β5t^+^ progenitors and mature mTEC, based on their progressive age-related quiescence and limited differentiation into the medulla lineage (*12*).

Here, we seek to resolve the relationship between postnatal TEPC and mature TEC lineages. Using an inducible lineage-tracing system that depends on β5t expression, we uncover the early kinetics of TEPC-mTEC progenitor-progeny relationships and resolve the contribution of these cells to cTEC, mTEC, and intertypical TEC subsets. Moreover, by investigating the earliest stages of TEPC-TEC differentiation and lineage tracing with single-cell transcriptomics, we identify two candidate lineage-biased progenitor subtypes delineated by the expression of lymphocyte antigen 6 family member D, encoded by *Ly6d*, that are spatially segregated in the thymus. Last, we note the disproportionate impact of aging on *Ly6d*^−^ versus *Ly6d*^+^ progenitors, which has consequences for rejuvenation of the aging thymic epithelium.

## RESULTS

### mTEC-biased precursors express β5t

Postnatal mTECs are derived from epithelial precursors that express the proteasome subunit β5t encoded by the *Psmb11* gene (*39*). Aging reduces the differentiation efficiency of β5t^+^ progenitors, raising the possibility that additional TEC progenitor cells with different developmental capacities could contribute to the maintenance of the postnatal thymus epithelium (*12*, *18*, *30*). To explore this possibility, we used a previously described lineage tracing model to track the progeny of β5t-expressing TECs. This mouse model, designated 3xtg^β5t^, relies on *Psmb11* promoter activity to drive expression of the reverse tetracycline transactivator (rtTA) (*18*, *30*). Administration of doxycycline (Dox), therefore, leads to Cre expression, the removal of a stop-cassette, and subsequent expression of the fluorescent reporter ZsGreen (fig. S1A). Previous observations show that this transgenic model also labeled a small fraction (1 to 2%) of lineage committed mature mTECs, a finding attributed to promiscuous expression of the *Psmb11* locus (*18*, *42*).

**Fig. 1.**
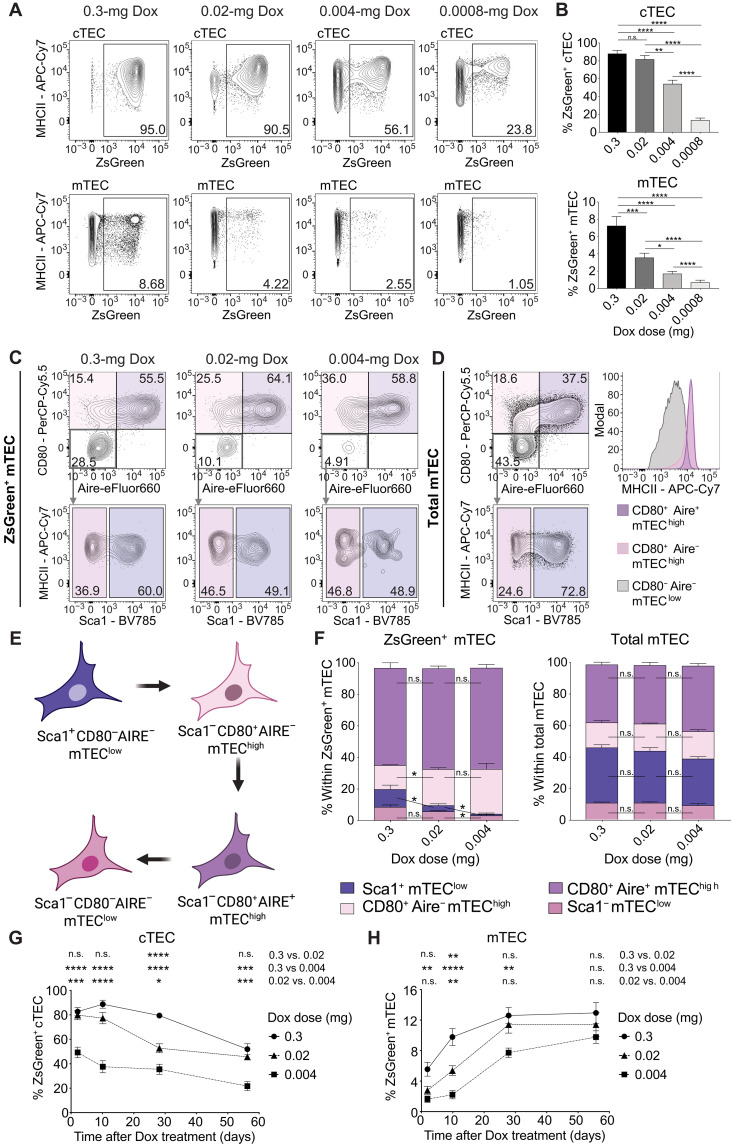
mTEC derived from a β5t^+^ progenitor accumulate in the first 9 weeks of life. (**A**) Representative flow cytometry plots and (**B**) quantification of ZsGreen^+^ cTECs (top) and mTECs (bottom) in mice injected with decreasing doses of Dox 2 days prior. *n* = 11 mice per group, except for the 0.0008-mg dose which consisted of *n* = 6 mice. (**C** and **D**) Representative flow cytometry plots of ZsGreen^+^ mTEC (C) and total mTEC (D) depicting expression of MHC-II, CD80, AIRE, and Sca1 2 days after Dox treatment (0.004 mg). Flow cytometry gating on MHCII expression leads to mTEC^low^ and mTEC^high^ phenotypes, as in (C) and (D). (**E**) Schematic representation of mTEC subpopulation lineage relationships. Created in BioRender. Morgan, M. (2026) https://BioRender.com/060emei. (**F**) Quantification of the relative proportions of the different ZsGreen^+^ mTEC (left) and total mTEC (right); *n* = 6 mice per group, from two independent experiments. Statistical significance was determined by multiple unpaired Welch’s *t* tests; *P < 0.05, **P < 0.01, ***P < 0.001, and ****P < 0.0001. (**G** and **H**) Relative proportions of ZsGreen-labeled (G) cTEC (Ly51^+^UEA1^−^) and (H) mTEC (Ly51^−^UEA1^+^) over the course of 8 weeks post–Dox treatment with decreasing doses. *n* = 11 mice per time point and treatment group, except for 56-day time point of the 0.3-mg dose with *n* = 10. Statistical significance was determined by mixed-effects model, using the maximum likelihood method. Correction for multiple comparisons was performed using the Tukey method, and *P* values were adjusted accordingly. The results from the multiple comparisons are depicted as follows: *P < 0.05, **P < 0.01, ***P < 0.001, and ****P < 0.0001. Error bars represent the SEM. n.s., not significant.

We took advantage of this model to study differential fate choices of mTECs from β5t^+^ or β5t^−^ progenitors. We first investigated whether decreasing Dox doses would reduce the labeling of mature mTEC while maintaining the marking of TEC precursors and their immediate progeny (see fig. S2 for full gating strategy). Treatment of 3xtg^β5t^ mice at 7 days of age with a single dose of either 0.3, 0.02, 0.004, or 0.0008 mg of Dox labeled a decreasing number of TECs in a dose-dependent fashion (Fig. 1, A and B; and fig. S1, C and D). The lowest dose (0.0008 mg) resulted in a very limited labeling across all TEC populations (<10%) and was, therefore, not further considered. Analyzed 2 days after the Dox pulse (Fig. 1, C to H), most of cTECs were labeled because of their constitutive expression of *Psmb11* (Fig. 1, A and B, top) (*43*). In contrast, only a small fraction of mTEC was labeled at this time point (0.3 mg, 7.20 ± 3.57%; 0.02 mg, 3.52 ± 1.74%; and 0.004 mg, 1.66 ± 0.97%; Fig. 1, A and B, bottom), most of which had a mature CD80^high^Aire^+^ phenotype (0.3 mg, 76.88 ± 4.2%; 0.02 mg, 86.78 ± 3.85%; and 0.004 mg, 92.57 ± 4.40%; Fig. 1, C to F; and fig. S3, A to C), a result attributed to the promiscuous expression of β5t. In addition, higher doses of Dox preferentially labeled lineage immature Sca1^+^CD80^−^AIRE^−^MHCII^low^ mTECs without any apparent drug toxicity (Fig. 1, C to G; and fig. S3, A to C). Therefore, we found that the main differences among Dox doses with regard to mTEC labeling were not due to differential labeling of mature mTECs, but rather to a dose-dependent labeling of immature Sca1^+^CD80^−^AIRE^−^MHCII^low^ mTEC.

To investigate the long-term dynamics of TEC progenitor labeling, we studied 3xtg^β5t^ mice 10, 28, and 56 days after Dox treatment. Our analysis revealed differential labeling dynamics across individual TEC subpopulations. For example, the frequency of labeled cTEC decreased over time by ∼20%, which was independent of the Dox dose used (Fig. 1H). In contrast, the frequency of labeled mTECs increased over the same time frame, resulting in equivalent proportions of labeled cells across the different Dox doses after 8 weeks (Fig. 1H). These data both confirmed the contribution of β5t^+^ postnatal progenitors to the mTEC lineage and suggested that any of the Dox doses used in our experiments were similarly effective in labeling mTECs long term. Therefore, to minimize the potential confounding effects of labeling immature Sca1^+^CD80^−^Aire-MHCII^low^ mTEC, we used a single Dox dose of 0.004 mg for all subsequent lineage-tracing experiments.

### Developmental dynamics of TEC differentiation

**Fig. 2.**
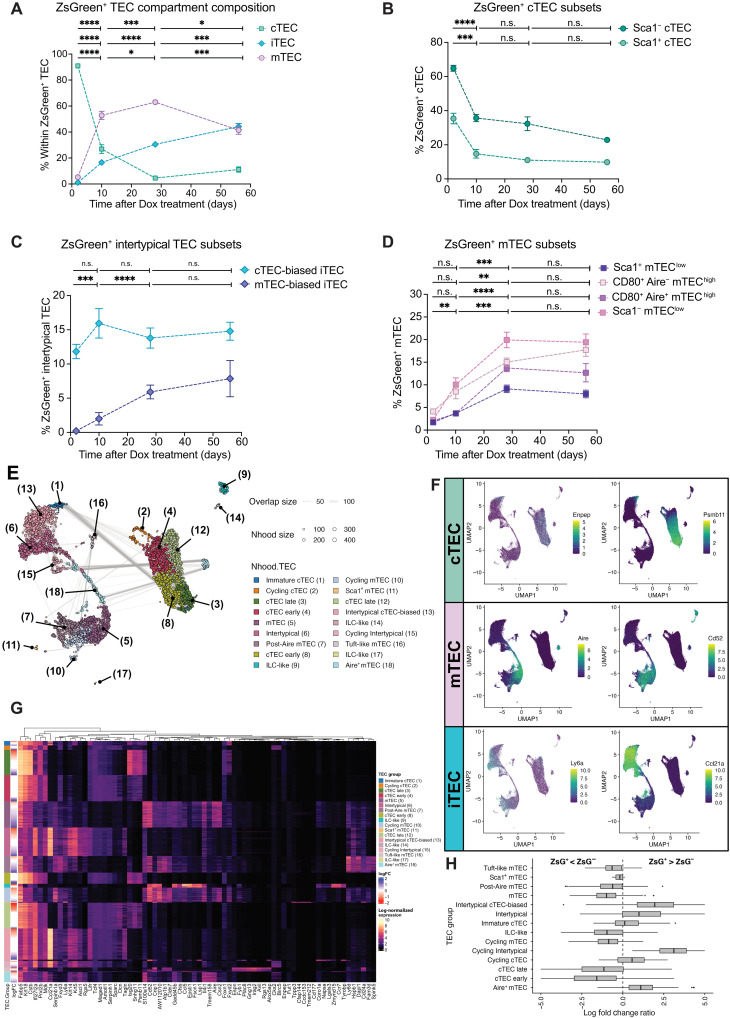
Differential progenitor-progeny relationships are revealed by lineage tracing and scRNA-seq. (**A** to **D**) Analysis of ZsGreen^+^ TEC compartment composition among (A) total TECs, (B) cTECs, (C) intertypical TECs, and (D) mTECs following intraperitoneal injection of 0.004 mg Dox at 7 days of age and a chase period of 2, 10, 28, and 56 days. *n* = 7 mice for the analysis at day 2 and *n* = 8 for that at days 10, 28, and 56 after Dox treatment. Error bars represent the SEM. Statistical significance was determined by using multiple *t* tests to calculate differences in the TEC subset composition between pairs of consecutive time points. Correction for multiple comparisons was performed using the Holm-Šídák method. *P < 0.05, **P < 0.01, ***P < 0.001, and ****P < 0.0001. (**E**) Neighborhoods anchored by single-cell uniform manifold approximation and projection (UMAP) coordinates illustrating TEC heterogeneity. Points represent neighborhoods with UMAP coordinates defined by the index cell for each. Colors denote TEC subtype annotations based on marker genes [see (G)]. Point size displays the number of cells in a neighborhood, and connecting lines represent the number of overlapping cells between each pair of neighborhoods. Numbers are annotated for each corresponding cluster. (**F**) UMAP of postnatal EpCAM^+^ single-cell transcriptomes. Each panel is colored by markers of TEC lineages: cTECs (*Enpep*, which encodes Ly51; and *Psmb11*), mTECs (*Aire* and *Cd52*), and intertypical TECs (*Ccl21a*; and *Ly6a*, which encodes Sca1). (**G**) Heatmap of TEC subpopulation marker genes. TEC subtype clusters are in the same order as the figure legend. (**H**) Log fold change ratio (*x* axis) across annotated neighborhood group (*y* axis). Box plots show the median LFC ratio, and box edges are the lower and upper quartiles with whiskers extending to 1.5× the interquartile range (IQR). Outliers beyond this range are denoted as individual points. n.s., not significant.

To gain insight into the temporal dynamics of TEC progenitor-progeny relationships, we examined how the frequency of labeled subpopulations changed during the 8 weeks following Dox treatment of 7-day-old mice (Fig. 2A). In agreement with our previous findings (*12*), cTECs and mTECs contributed relatively equally to the total TEC number early in life (day 2 post–Dox treatment: cTEC, 39.8 ± 4.2%; and mTEC, 37.7 ± 2.9%; fig. S4A). By 8 weeks (56 days) post–Dox treatment, mTECs comprised a larger fraction of labeled TEC compared to cTEC phenotypes [see below day 56 post-Dox treatment; cTEC (6.5 ± 2.1%) versus mTEC (35.6 ± 5.7%); Fig. 2, A and B]. In parallel, the frequency of intertypical TECs, which we defined based on the coexpression of CCL21 and Sca1, increased progressively over the same period (day 2 post–Dox treatment, 18.2 ± 2.6%; week 5, 43.9 ± 1.7%; and day 56 post–Dox treatment, 56.2 ± 3.4%; figs. S4A to S6).

We further examined the contribution of TEC progenitors to cTECs, mTECs, and intertypical TECs, assessed by the frequency of ZsGreen^+^ cells in each of these subpopulations over the indicated time course (Fig. 2, A to D). The initial high frequency of ZsGreen^+^ cTECs (day 2 post–Dox treatment, 91.1 ± 1.5%) decreased rapidly over time, reaching a low level 4 weeks after Dox treatment, which remained largely unchanged thereafter (day 28 post–Dox treatment, 11 ± 5.6%; Fig. 2A and fig. S6A). cTECs can be further classified into two separate subpopulations on the basis of their MHCII and Sca1 expression. Based on these two markers, MHCII^hi^Sca1^−^ cTEC (designated cTEC^hi^) would represent a mature cTEC population, whereas MHCII^low^ Sca1^+^ (cTEC^lo^) have been proposed to constitute a more immature cTEC state (*44*). cTEC^hi^ were highly abundant in 9-day-old mice (day 2 post–Dox treatment), but their frequency reduced over time and matched that of cTEC^lo^ in 9-week-old animals (day 56 post–Dox treatment; fig. S4B). These results are consistent with normal thymic postnatal dynamics (*12*). Although the percentage of labeled cells differed between the two cTEC phenotypes, the decrease of ZsGreen positivity over time was comparable (Fig. 2B), rendering a precursor-progeny relationship between these two cTEC subpopulations unlikely.

The frequency of intertypical TECs as a fraction of all TECs increased threefold over the time course, whereas the proportion of ZsGreen^+^ cells among intertypical TECs increased almost 50-fold [day 2 post–Dox treatment (0.96 ± 0.54%) versus day 56 post–Dox treatment (44.1 ± 6.7%)] (Fig. 2A and fig. S4A). Based on similar aging dynamics, we previously hypothesized that intertypical TECs accumulate in a lineage-biased state that restricts their differentiation into mature mTEC (*12*). To investigate the lineage bias of intertypical TEC, we used UEA1 and Ly51, respectively, to identify any skewing to either a medullary or a cortical TEC phenotype (Fig. 2, B and C, and fig. S4C). We observed that the frequency of ZsGreen^+^ cTEC-biased intertypical TECs (Ly51^+^CCL21^+^Sca1^+^UEA1^−^) remained relatively stable over the 56 days of our time course (Fig. 2C). In contrast and over the same time, we observed a sustained accumulation of ZsGreen^+^ mTEC-biased intertypical TEC (Ly51^−^CCL21^+^Sca1^+^UEA1^+^; Fig. 2C). Our results show that the labeling kinetics of progeny derived from β5t^+^ progenitors shifts postnatally and preferentially gives rise to mTEC lineage-biased precursors (Fig. 2C).

Based on this observation, we next sought to trace the potential of ZsGreen^+^ mTEC-biased intertypical TEC. To achieve this, we reclassified mTECs into sequential stages of ontologically separate subpopulations on the basis of MHCII, Sca1, CD80, and Autoimmune regulator (AIRE) expression (Fig. 1E). In this scheme, MHCII^low^Sca1^+^CD80^−^AIRE^−^ mTECs represent an immature stage that differentiates into a mature (MHCII^high^Sca1^−^CD80^+^AIRE^−^) subpopulation via a MHCII^high^Sca1^−^CD80^+^AIRE^−^ transitional state. Mature (MHCII^high^Scal1^−^CD80^+^Aire^−^) mTECs lastly give rise to a terminally differentiated subpopulation of MHC^low^Sca1^−^CD80^−^AIRE^−^ mTEC (*20*, *44*–*46*). Over time, the frequencies of all but the least mature mTEC decreased (fig. S4D). However, the proportion of ZsGreen^+^ cells increased progressively up to 4 weeks after labeling, whereupon their relative frequency plateaued (Fig. 2D). These dynamics suggest that mTEC-biased ZsGreen^+^ intertypical TECs eventually contribute to all stages of mTEC maturity. However, these observations could also be explained by the biased expansion of a particular subpopulation of ZsGreen^+^ intertypical TECs (which have not yet been further identified using convenient cell surface markers) or mTECs. To exclude this possibility, we determined the proliferation rate of TEC subpopulations 2 days post–Dox treatment in relation to their ZsGreen-labeling status. We found that ZsGreen^+^ cells were less proliferative than unlabeled cells, as measured by Ki67 staining (fig. S4, E to G), ruling out biased proliferation in ZsGreen^+^ cells.

In summary, our results indicated that β5t^+^ progenitors progressively contributed to each of the TEC lineages examined here. Moreover, the inheritance of ZsGreen^+^ label from postnatal β5t^+^ mother to daughter cells suggested that labeled intertypical TEC preferentially gave rise to mTEC and that medulla lineage-biased intertypical TEC accumulated progressively over the first 9 weeks of the life course, a finding in agreement with our previous observations in aged mice (*12*). Our results further exclude a continuous contribution of β5t^+^ cTEC precursors into the intertypical TEC pool. Last, the large reduction in ZsGreen^+^ cTEC indicates a rapid turnover of these cells early in the postnatal mouse thymus despite the constitutive expression of *Psmb11* in the cortex (*22*, *46*).

### TEC lineage progenitors differentially accumulate in the first weeks of life

The temporal analysis of ZsGreen-labeled Ly51^−^UEA1^+^ cells showed as early as 2 days post-Dox that most cells displayed a mature mTEC phenotype (MHC^high^Sca1^−^CD80^+^) in accordance with the promiscuous expression of *Psmb11* (fig. S3D). In contrast, 10 days was the earliest time point when an appreciable fraction of immature (MHCII^low^Sca1^+^ CD80^−^AIRE^−^) and post-Aire (MHC^low^Sca1^−^CD80^+^AIRE^−^) phenotypes was labeled (fig. S3D). Therefore, to further investigate the early steps in progeny-progenitor relationships, we combined our inducible lineage tracing system with single-cell gene expression profiling at 2 and 10 days post–Dox treatment. On the basis of our and others’ recent findings (*12*, *14*), we reasoned that a progenitor TEC might express protein marker characteristics of a combined lineage phenotype (bipotent) or alternatively, restricted to either the mTEC or cTEC-specific lineages (unipotent), respectively. We therefore sorted and analyzed equal numbers of both ZsGreen^−^ and ZsGreen^+^ cTEC and mTEC. To prevent batch effects confounding our analysis, we stained the sorted cells with hashtag oligonucleotide (HTO)–labeled antibodies and multiplexed across individual ZsGreen fractions and time points before droplet scRNA-seq. Following quality control (fig. S7), we integrated single-cell gene expression profiles across three replicate experiments for each ZsGreen fraction and time point from 64,500 high-quality cells. Visualizing marker gene expression and cellular indexing of transcriptomes and epitopes by sequencing (CITE-seq) proteins MHCII, CD86, Sca1, and CD24 (fig. S8) on a low-dimensional embedding of single-cell transcriptomes using uniform manifold approximation and projection (UMAP) indicated the presence of multiple TEC lineages (Fig. 2, E and F, and fig. S8). These included mature cTECs (*Cxcl12*, which encodes C-X-C motif chemokine 12; and *Psmb11*), mature mTECs (*Cd52*, which encodes the glycoprotein CAMPATH-1 antigen; and *Aire*), and intertypical TECs (*Ccl21a*; and *Lifr*, which encodes leukemia inhibitory factor receptor subunit alpha) (*12*). Because single-cell gene expression measurements are noisy and sparse, we constructed a set of *k*-nearest neighbor (kNN) graph neighborhoods using Milo (*47*), which represents the single-cell data using overlapping cell states (neighborhoods).

Clustering similar TEC neighborhoods yielded 18 clusters, which we annotated on the basis of canonical and cluster-specific marker gene expression profiles (Fig. 2, E to G, and fig. S8). This analysis revealed most of previously described adult mouse TEC lineages (*12*), including Tuft-like mTEC (cluster 16) and post-Aire mTEC (cluster 7). This analysis also identified innate lymphoid cells that displayed Epithelial cell adhesion molecule (EpCAM) on their surface, but in which the corresponding mRNA was not detected (fig. S9). These cells were excluded from further analysis. The transcriptional similarity between cells positioned far apart in the UMAP space was more illustrated by visualizing the neighborhood graph (Fig. 2E), which resolved the connection between immature cTEC (cluster 1) and early cTEC (cluster 8) and between intertypical TEC (cluster 6) and Aire^+^ mTEC (cluster 18). Notably, our clustering analysis indicated multiple neighborhood clusters that corresponded to intertypical TEC, which we previously hypothesized may contain TEC progenitors based on shared gene expression profiles e.g., *Ccl21a*, *Ly6a*, and *Lifr* (Fig. 2G) (*12*).

To identify transcriptional TEC cell states that had different abundance between day 2 and day 10 post–Dox treatment, we used differential abundance (DA) testing algorithm Milo (*47*, *48*), analyzing ZsGreen^+^ and ZsGreen^−^ fractions separately [10% false discovery rate (FDR)]. Considering just the ZsGreen^+^ cells, our analysis revealed across the 8-day time course an enrichment of intertypical TECs with a cTEC-biased transcription profile (cluster 13; *Cxcl12*, *Ccl21a*, *Krt5* that encodes keratin 5, and *Krt14* that encodes keratin 14; fig. S9), a finding concordant with our flow cytometry data (Fig. 2C) and the low but detectable expression of *Psmb11* in these cells (Fig. 2F).

We next investigated the lineage biases in each neighborhood cluster of ZsGreen^+^ and ZsGreen^−^ TEC. To do this, we computed the ratio of the log fold changes from the separate ZsGreen^+^ and ZsGreen^−^ Milo analyses for each neighborhood and grouped them by TEC neighborhood cluster. This analysis showed an accumulation of ZsGreen positivity in cycling cTEC-biased intertypical TEC, as well as Aire^+^ mTEC (Fig. 2H), findings that are consistent with our flow cytometry data (Fig. 2, A to D). Also consistent with that analysis was the observation of a depletion of ZsGreen^+^ cTEC populations, reflecting the relatively high postnatal turnover of these TEC subtypes (Fig. 2, B and H) (*49*). In contrast, the neighborhoods representing the remaining mTEC subtypes, including post-Aire, Tuft-like, and cycling mTECs, were skewed toward ZsGreen negativity (Fig. 2H).

A transit-amplifying population of mTECs has previously been described and proposed as the progenitor pool of postnatal mTEC, given the name transit amplify cell-TEC (TAC-TEC) (*50*). In our data, we identified three clusters of dividing cells: cTEC cluster 2, mTEC cluster 10, and intertypical TEC cluster 15. To assess whether any of the proliferating TEC clusters identified here correspond to TAC-TEC, we integrated the two single-cell transcriptomic datasets (fig. S11). The cross-dataset mapping indicated that TAC-TEC best corresponds to a mixed subset of intertypical TEC cluster 6, cycling intertypical TEC (cluster 15), and Aire^+^ mTEC (cluster 18). This heterogeneity has been noted previously (*50*), and our improved separation of dividing TEC subtypes is likely explained by the larger number of cells in our data. Notably, cycling intertypical TECs (cluster 15) were strongly enriched in the ZsGreen^+^ fraction (Fig. 2H), which suggests that either they arise from or contain a β5t^+^ progenitor state or the labeling efficiency is specifically skewed in dividing cells (fig. S4F); Ki67 staining ruled out the latter explanation (fig. S4F). Moreover, as cycling intertypical cells appeared to form a transitional population between intertypical TEC and Aire^+^ mTEC and because intertypical TEC also accumulated the ZsGreen label, our results further support the notion that, rather than TAC-TEC containing the mTEC progenitor, they are grouped into intertypical TEC cluster 6.

In summary, our neighborhood analysis of perinatal TEC development and progenitor-progeny relationships are consistent with at least one β5t^+^ progenitor. Our results do not delineate between a single bipotent β5t^+^ progenitor and two discrete unipotent β5t^+^ TEPCs that give rise to the cTEC and mTEC lineages separately. However, our combined flow cytometry and single-cell data strongly support multiple progenitor populations that share characteristics with intertypical TECs, each likely displaying distinct TEC lineage biases (Fig. 2, E to G).

### Ly6d expression distinguishes lineage-biased TEC progenitors

**Fig. 3.**
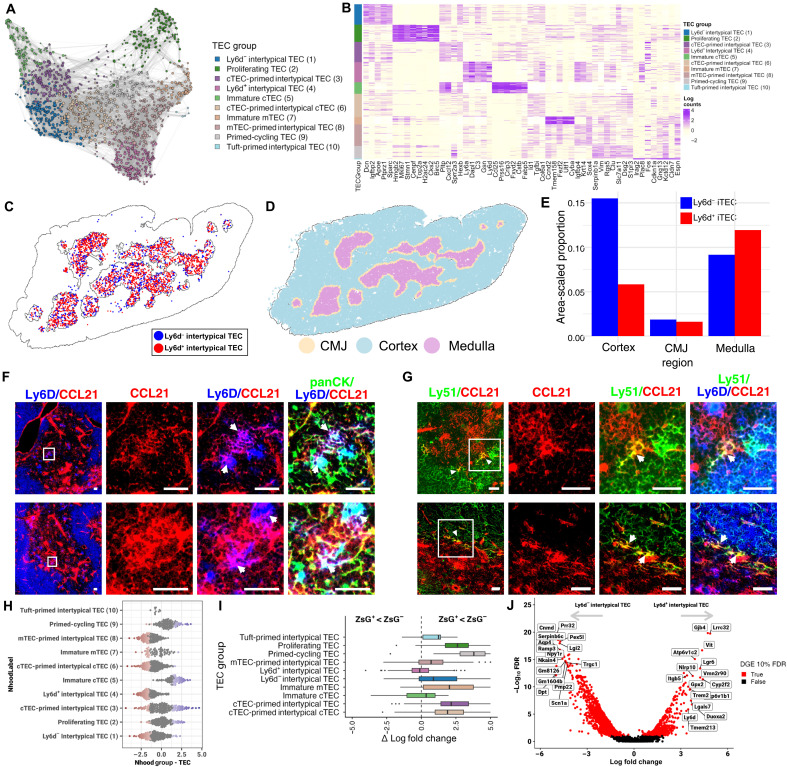
Stratification of progenitor TEC and differential lineage labeling over time. (**A**) UMAP of intertypical TEC neighborhoods (1466 neighborhoods). Points are the neighborhood index cells embedded in the UMAP of intertypical TECs (fig. S13), colored by neighborhood cluster. Edges represent the number of cells shared between neighborhoods, and point sizes denote the number of cells in each. (**B**) Heatmap of expression of top marker genes for clusters shown in (A). Rows denote neighborhoods and columns are genes. (**C**) Spatial positions of *Ly6d*^+^ and *Ly6d*^−^ intertypical TECs. Each point represents a segmented single intertypical TEC (*Ccl21a*^+^ *Lifr*^+^). (**D**) All cells are labeled by inferred thymic compartment: cortico-medullary junction (CMJ; yellow), cortex (turquoise), or medulla (pink). (**E**) Bar chart of the compartment area–normalized proportion (*y* axis) of *Ly6d*^+^ (red) and *Ly6d*^−^ (blue) intertypical TEC within spatial compartments (*x* axis) as in (D). (**F** and **G**) Immunofluorescence images of male thymic lobes stained with anti-Ly6D (blue), anti-CCL21 (red), and (F) anti-panCK or (G) anti-Ly51 (green). Right panels are close-ups of the tissue area outlined by white boxes in the top and bottom far-left panels. White arrows denote TECs that are (F) CCL21^+^Ly6D^+^PanCK^+^ and (G) CCL21^+^Ly6D^−^Ly51^+^. Scale bars, 50 μm. (**H**) Beeswarm plot of DA neighborhoods between ZsGreen^+^ and ZsGreen^−^ samples. Points are colored by log fold change (*x* axis). (**I**) Box plot of ΔLFC between ZsGreen^+^ and ZsGreen^−^ DA neighborhoods (10% FDR). Box plot horizontal bars denote the median ΔLFC, box margins are the IQR, with whiskers extending to 1.5× the IQR; outliers are shown as individual points. Plot colors are as in (A). (**J**) Volcano plot of differentially regulated genes between *Ly6d*^+^ (cluster 4) and *Ly6d*^−^ (cluster 1) intertypical TEC. Statistically significant differentially expressed genes (DEGs; 10% FDR) are colored in red. Labeled genes denote the top 15 up- and down-regulated genes. DGE, differential gene expression.

To further investigate the possibility that the population of intertypical TEC includes one or more potential progenitor populations, we restricted our single-cell analysis to data corresponding to the least mature TECs (i.e., clusters 1, 6, 13, and 15). This selection was supported by cross-mapping to scRNA-seq data of human TECs (fig. S12) (*51*). Human cells corresponding to a poly-keratin signature [described by Ragazzini *et al.* (*51*) as human neighborhood cluster 7] had the highest transcriptional correlation with intertypical TECs and immature mTECs and cTECs in our data (fig. S12). Therefore, we constructed a kNN graph with a newly computed set of highly variable genes (HVGs) and reduced dimensions that captured the gene expression variation across the cells from clusters 1, 6, 13, and 15 (19,956 cells, fig. S13). We then identified marker genes for a new set of 10 neighborhood clusters and manually annotated these cells (Fig. 3, A and B). Our higher resolution analysis revealed a heterogeneous mix of TEC subtypes that expressed genes characteristic of intertypical TEC, mTEC, and cTEC lineages (Fig. 3, A and B; fig. S14, and table S1). The immature mTEC (cluster 7) and cTEC states (cluster 5) were broadly separable in our neighborhood graph, delineated by lineage markers (cTEC: *Ccl25* that encodes C-C motif chemokine ligand 25, *Prss16* that encodes the thymoproteasomal protein thymus-specific serine protease, and *Psmb11*; mTEC: *Fezf2* that encodes the transcription factor FEZ family zinc finger 2 and *Aire*). In addition, our neighborhood graph suggested that an mTEC fate choice was made before cell division, indicating that previously described TAC-TEC cells may already be committed to the mTEC lineage (Fig. 3A and fig. S11).

We identified two intertypical TEC neighborhood subclusters (1 and 4), which expressed the highest levels of genes previously described as markers for TEC progenitors (*Gas1* that encodes growth arrest-specific protein 1, *Ly6a*, *Itga6* that encodes integrin alpha 6 protein, and *Plet1* that encodes the placenta expressing transcript 1 protein; fig. S14) (*21*, *29*) but differed in their expression of *Ly6d* that encodes lymphocyte antigen 6 family member D (Fig. 3B and fig. S14). The *Ly6d*^+^ intertypical TECs displayed a pattern of lowly expressed genes shared with mTEC subclusters 7 and 8 (e.g., *Cd52*, *Aire*, and *Fezf2*; fig. S13) and expressed transcription factor genes associated with type I interferon signaling (*Stat1* and *Stat2* that encode signal transducer and activator of transcription 1 and 2, respectively; and *Irf6* that encodes interferon regulatory factor 6; fig. S15). Recent data show that interferon signaling is critical for mTEC maturation during human fetal thymus development (*52*), indicating that *Ly6d*^+^ intertypical TECs might be precursors of, or biased toward, the mTEC linage. In contrast, the *Ly6d*^−^ intertypical TECs shared gene expression patterns with cTEC subclusters 3 and 5, including *Dll4* (encoding Delta-like ligand 4) and *Pdpn*, and are characterized by transcription factors associated with differentiation and stem cells [e.g., *Id3* that encodes inhibitor of DNA bind 3, *Nfib* that encodes nuclear factor 1 B type, and *Peg3* (paternally expressed 3) that is an imprinted gene in humans; fig. S15] (*53*)*.* We further identified five subclusters that we call primed intertypical TEC, so called because they share transcriptional profiles with intertypical TEC and either mTEC (cluster 8), cTEC (clusters 3 and 6), Tuft-like mTEC (cluster 10), or proliferating TEC (cluster 9; Fig. 3, A and B, and fig. S14). We speculate that these cells may be in the initial stages of differentiation or making a fate choice toward a particular lineage or the cell cycle. Last, we note that *Psmb11* is highly expressed in the immature cTEC subcluster 5 and more lowly expressed in the cTEC-primed intertypical TEC and Ly6d^−^ intertypical TECs (fig. S14). Together, these analyses suggest the presence of up to two possible progenitor TEC states, where their lineage bias is delineated by the expression of *Ly6d*.

To explore how *Ly6d* expression marks cortical versus medullary biased progenitors, we mapped the marker gene profiles for each onto single-cell spatial transcriptomics profiles (10x Genomics Xenium) of an 8-week-old thymus (Fig. 3, C and D; see Materials and Methods). Using marker genes identified by differential gene expression analysis, we computed a combined module score to classify each intertypical TEC (*Ccl21a*^+^*Lifr*^+^) in our spatial transcriptomics dataset as either *Ly6d*^+^ or *Ly6d*^−^ intertypical TEC (fig. S16, A to D). Remapping the module score back onto our original scRNA-seq data using the same genes validated that this approach was selective for *Ly6d*^+^ and *Lyd6*^−^ intertypical TEC (fig. S16, E and F). Our spatial analysis showed that *Ly6d*^+^ intertypical TECs were enriched within the medullary regions, whereas the *Ly6d*^−^ intertypical TECs were more likely to be present in the CMJ region or adjacent in the cortex (Fig. 3E and fig. S16G). Immunofluorescence staining for proteins CCL21 and Ly6D and either panCK in the medulla or Ly51 in the cortex corroborated the localization of *Ly6d*^+^ and *Ly6d*^−^ to specific thymic compartments (Fig. 3, F and G). Together, these complementary data demonstrate that two intertypical TEC populations, delineated by *Ly6d* expression, are both spatially segregated and characterized by transcriptome profiles indicative of different TEC lineages.

Given the different spatial compartmentalization of *Ly6d*^+/−^ intertypical TECs, we wanted to understand their lineage relationship to β5t^+^ progenitors. To do this, we probed the composition of the ZsGreen^+^ and ZsGreen^−^ fractions within the intertypical and immature TEC states in our scRNA-seq data. Using Milo (*47*, *48*), our analysis revealed that immature cTECs, cTEC-primed intertypical TECs (subcluster 3), and both primed-cycling (subcluster 9) and proliferating TECs (subcluster 2) were enriched within the ZsGreen^+^ fraction (10% FDR, Fig. 3H). In contrast, *Ly6d*^−^ intertypical TECs (subcluster 1), *Ly6d*^+^ intertypical TECs (subcluster 4), and mTEC-primed intertypical TECs (subcluster 8) were depleted in that fraction. This analysis indicated that both *Ly6d*^−^ and *Ly6d*^+^ intertypical TECs may not arise from a β5t^+^ progenitor state, irrespective of their apparent lineage biases. To explore this further, we used Milo to compare the abundance of TEC neighborhoods between 2- and 10-day time points following Dox treatment (10% FDR; fig. S17). Over the course of 8 days, both *Ly6d*^+^ and *Ly6d*^−^ intertypical TECs were enriched over time regardless of whether these cells were ZsGreen positive or negative (fig. S17). Additionally, we observed for the ZsGreen^+^ fraction a relative enrichment of primed-cycling TECs (subcluster 9) and cTECs (subcluster 5; fig. S17), and a corresponding lower frequency of cTECs was noted at 10 days within the ZsGreen^−^ fraction. We reasoned that, if intertypical TECs either contained or were the progeny of β5t^+^ progenitors, then a relative accumulation of ZsGreen^+^ cells should be observed over time. Conversely, should either one or both intertypical TEC populations precede the developmental stage of β5t^+^ progenitors, a change in the ZsGreen fractions would not occur. Therefore, we computed for each neighborhood the difference between the day 2 versus day 10 log fold changes for the ZsGreen^+^ and ZsGreen^−^ fraction of TEC [Δ log fold change (ΔLFC); Fig. 3I]. Our analysis revealed that cTEC-primed intertypical TECs (subclusters 3 and 6) accumulated over time in the ZsGreen-labeled fraction, just like proliferating (subcluster 2) and primed-cycling TECs (subcluster 9). The latter are TECs with a transcriptional profile concordant with both dividing cells (*Mki67* that encoded Ki67, *Top2a* that encodes DNA topoisomerase II alpha, and *Ccnd2* that encodes cyclin D2) and an mTEC-like expression profile (*Cd52*, *Cd40*, and *Aire*; fig. S14). While there was a higher percentage of *Ly6d*^−^ intertypical TECs accumulating in the ZsGreen^+^ fraction, this was not observed for *Ly6d*^+^ intertypical TECs (Fig. 3I), suggesting that *Ly6d*^+^ intertypical TECs may arise from an unlabeled progenitor or, alternatively, precede a state of β5t positivity.

Although ZsGreen does not preferentially accumulate in *Ly6d*^+^ intertypical TECs over our time course, we observed a small fraction that were ZsGreen positive despite a lack of detectable *Psmb11* expression (fig. S13). We hypothesized that this may be due to a stochastic activation of the *Psmb11* promoter. To explore this premise further, we correlated the proportion of ZsGreen^+^ cells in each neighborhood with the proportion that expresses TEC progenitor and lineage genes (cortex: *Psmb11*, *Prss16*, and *Ccl25*; medulla: *Aire* and *Fezf2*; progenitor: *Ly6a*, *Ccl21a*, and *Ly6d*; fig. S18A). This analysis revealed that the proportion of ZsGreen^+^ Ly6d^+^ intertypical TECs was positively, albeit weakly, correlated with the proportion of *Foxn1^+^* {ρ = 0.20, 95% confidence interval (CI) [0.06 to 0.33]} and *Psmb11*^+^ cells (ρ = 0.28, 95% CI [0.15 to 0.41]). Correlations between ZsGreen expression and other Foxn1-regulated genes (*54*) were notably weaker in comparison (*Ccl25*: ρ = 0.12, 95% CI [−0.02 to 0.26]; and *Prss16*: ρ = 0.12, 95% CI [−0.02 to 0.26]). These correlations appeared to be driven by a small subset of neighborhoods containing ≥50% ZsGreen^+^ and ≥25% *Foxn1*^+^ cells, because the number of *Psmb11^+^* cells was low (mean 0.72%; fig. S18B). Therefore, weak activation of the *Psmb11* promoter during Dox treatment in *Foxn1*^+^ cells, despite the absence of detected *Psmb11* mRNA transcripts, may lead to the labeling of some TECs that are not the progeny of a β5t^+^ progenitor.

### Differential signaling cues between Ly6d^+^ and Ly6d^−^ intertypical TECs

To understand the extracellular signals and gene regulatory programs that define the *Ly6d^+^* and *Ly6d*^−^ intertypical TECs, respectively, we first performed differential gene expression and functional enrichment analyses. We compared gene expression levels between *Ly6d^+^* and *Ly6d*^−^ intertypical TECs across the mouse protein-coding transcriptome and identified 1992 differentially expressed genes (DEGs; 1% FDR, Fig. 3J). These included, in addition to *Ly6d*, the R-spondin receptor *Lgr6*, leucine-rich repeat containing 32 (*Lrcc32*), also known as glycoprotein A repetitions predominant (GARP), and galectin 7 encoded by *Lgals7*, which had previously been observed in Hassal’s corpuscles (*55*). This expression profile indicated both paracrine and cell-matrix interactions by *Ly6d*^+^ intertypical TECs. To gain a broader insight into the gene regulatory programs in each cell type, we performed gene set enrichment analyses (GSEAs; fig. S19). *Ly6d*^−^ intertypical TECs had up-regulated genes related to mitosis and cell cycle regulation (e.g., Myc targets, E2F targets, and G_2_-M checkpoint), indicating the cells’ license for cell division and identifying them to be likely immediate progenitors of the primed-cycling TEC (subcluster 9). Retinol metabolism genes were enriched in the *Ly6d*^+^ intertypical TECs (fig. S19), which is required for in vivo and in vitro thymus organogenesis (*45*, *56*), suggesting an ongoing role for retinoic acid in postnatal TEC progenitors.

**Fig. 4.**
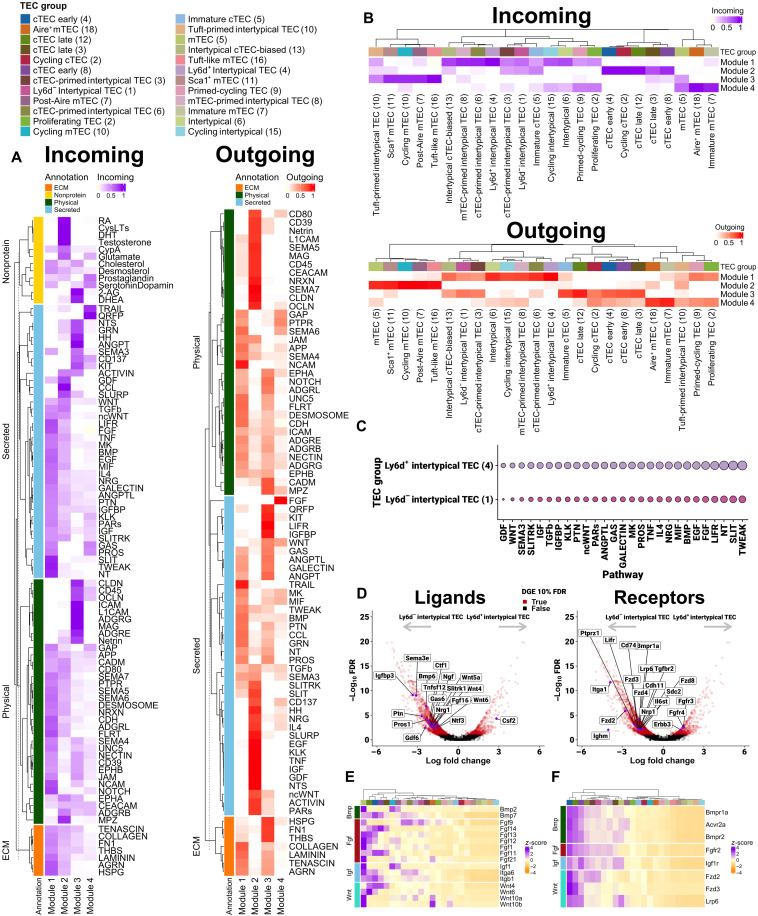
Cell-cell communication identifies TEC progenitor signaling cues. (**A**) Heatmap of NMF weights for each signaling pathway on coordinated modules. Each row is a CellChat signaling pathway for one of the four identified modules (columns). Each pathway is annotated on the basis of mechanism of action; either physical interaction (dark green), secreted paracrine (light blue), or extracellular matrix (ECM) interactions (orange). (**B**) Weighting of each TEC state on the coordinated signaling modules as in (A). (**C**) Module 1–specific signaling weights for *Ly6d*^+^ and *Lyd6*^−^ intertypical TECs. (**D**) Differential gene expression between *Ly6d*^+^ and *Ly6d*^−^ intertypical TECs highlights differential signaling cues. (**E** and **F**) Gene expression levels (*z*-scores; *y* axis) of differentially expressed (DE) signaling components shown as violin plots. Each row is a DE ligand (E) or receptor (F) for Wnt, Fgf, or Igf signaling receptor for TEC states (*x* axis). The color legend denotes the annotated TEC subtypes and corresponds to (B), (E), and (F).

On the basis of the observed differences in expression of cell-cell and cell–extracellular matrix interaction genes between *Ly6d*^+^ and *Ly6d*^−^ intertypical TECs, we next sought to identify the paracrine signaling pathways active in the postnatal thymic epithelium that could be important for TEPC. To achieve this, we first merged the annotation of all TEC clusters (Fig. 2E) with the higher resolution intertypical TEC subclusters (Fig. 3A and fig. S20A). Using the gene expression levels of this merged annotation as input to CellChat, we then identified 91 signaling pathways (fig. S20B), including Wnt, bone morphogenetic protein, and Notch that have known roles in thymus function, organogenesis, and TEC lineage differentiation (*44*, *57*–*61*). Using nonnegative matrix factorization (NMF) of the cell-cell communication network, we identified a complex landscape of incoming and outgoing signaling modules that were largely distinct for intertypical TECs, cTECs, and mTECs (Fig. 4, A and B). NMF decomposes the cell-to-cell communication network into the product of two weight matrices containing the signaling modules. The weights for each module represent the contribution of (i) each signaling pathway to the network and (ii) the contribution of each cell type; separate weights are estimated for incoming (receptor) and outgoing (ligand) signals.

Focusing first on the input signal module 1 that had the highest weight in *Ly6d*^+^ and *Ly6d*^−^ intertypical TECs, the ligand-receptor interactions with the greatest weighting included *Tnfsf12*-*Tnfrsf12a* (encoding Tweak-TweakR) and multiple Notch-Jag/Dll pairs: *Slit2*-*Robo1/2*, *Ntf5*-*Ngfr*, and *Lif*-*Lifr* (Fig. 4, A to C); *Lifr* is a marker gene of intertypical TECs (Fig. 2G). In addition, *Pros1* and *Gas6* share the receptor, *Axl*, and had high weight on incoming signal modules 1 and 4. The latter module was enriched in the mTEC lineage, suggesting that these pathways may be involved in mTEC versus cTEC fate choices or their lineage maintenance. Moreover, tumor necrosis factor (TNF) signaling (*Tnf*-*Tnfrsf1a*), which is known to promote TEC fate choices, was also strongly represented in module 1. The pattern of *Tnfrsf1a* expression, which encodes TNF receptor 1, was widespread across TEC subtypes, with lowest levels in mature, Tuft-like, and Aire^+^ mTEC and with highest levels in intertypical cTEC-biased and *Ly6d*^−^ intertypical TEC. *Tnfrsf1a* expression in *Ly6d*^+^ intertypical TECs was intermediate between *Ly6d*^−^ intertypical and mature mTECs (fig. S20C).

Module 2 was also enriched among *Ly6d*^−^ intertypical TEC and cTEC states, suggesting common signaling inputs. The ligand-receptor pairs with the highest weight on this module included testosterone, dihydrotestosterone, cysteinyl leukotrienes, retinoic acid, and *Ccl19/21/25-Ackr4*. Our single-cell data were derived from both male and female mice and are balanced across cell types and experimental conditions (fig. S21). Therefore, the observation that sex hormone signaling, which is a known early contributor to thymic involution (*62*), appears to differentially signal between TEC subtypes is interesting. There is a rapid turnover of cTECs early in mouse life (*46*), which we have confirmed in our experiments (Fig. 2, A and B, and fig. S9) and may therefore hint at a mechanism by which castration induces transient thymic renewal in male mice (*63*).

Last, incoming signal modules 3 and 4 had the highest weight in multiple mTEC subtypes, further illustrating the separation of cell-cell communication pathways between TEC lineages (Fig. 4B). The top five ligand-receptor interactions on module 3 involved adhesion molecules L1 adhesion molecule, G protein–coupled receptor G1, and intercellular adhesion molecule–1, as well as myelin-associated glycoprotein and angiopoietin 1 (Fig. 4A). In contrast, the top five incoming signals on module 4 were the neuropeptide and receptor *Qrfp-Qrfpr*, Trail-Trail receptor, prostaglandin via *Ptge3*-*Ptger3/4* and *Gas*-*Axl*, and serotonin/dopamine reception via *Htr1b* and *Htr7*. The highest expression for the receptors for these five paracrine and endocrine signals was in the Aire^+^ mTECs (fig. S21, A to E), suggesting that either they are involved in mTEC maturation or their expression is a consequence of promiscuous gene expression. To explore the latter, we reasoned that promiscuous gene expression would manifest as highly variable expression as opposed to constitutive expression within Aire^+^ mTEC. We compared the proportion of cells expressing top ligand-receptor genes from module 4 against a set of control mTEC genes (*Aire*, *Cd52*, *Epcam*, *Krt10*, *Fezf2*, and *Ascl1*) and Aire-dependent peripheral tissue antigen genes as identified by Sansom *et al.* (*42*). Our analysis showed that, while ligand and receptor genes were expressed heterogeneously across Aire^+^, mature, and post-Aire mTECs (fig. S21F), they were detected in a much higher proportion of cells than would be expected on the basis of promiscuous expression of peripheral tissue antigen genes.

In contrast to signal reception, we also examined the outgoing signaling from each TEC subtype, identifying a further four outgoing modules (Fig. 4, A and B). Like the incoming signals, we observed a separation of TEC subtypes by outgoing signaling modules. For instance, the outgoing module 1 that was enriched among intertypical TEC subtypes, independent of *Ly6d* expression, was strongly associated with TRAIL signaling (*Tnfs10*-*Tnfrsf10b*; Fig. 4A). The corresponding incoming signal was strongest among mTEC subtypes. TRAIL induces apoptosis upon binding to the TRAIL receptor, and a high rate of apoptosis has been described for immature mTEC as a potential quality control checkpoint during mTEC maturation (*55*). These findings suggest that progenitor TEC may signal to immature mTEC to regulate mature mTEC abundance.

To prioritize signaling cues important for TEC progenitor biology, we focused on the pathways with both strong incoming signals to the *Ly6d*^+^ and *Ly6d*^−^ intertypical TECs from module 1 (Fig. 4C) and that were differentially expressed between *Ly6d*^+^ and *Ly6d*^−^ intertypical TECs (Fig. 4, D and E). Notably, Wnt receptors *Fzd2*, *Fzd3*, *Fzd4*, and *Lrp6* were up-regulated in *Ly6d*^−^ intertypical TEC. Likewise, Wnt ligand genes *Wnt4*, *Wnt5a*, and *Wnt6*, implicating both canonical and noncanonical Wnt pathways, were up-regulated in *Ly6d*^−^ intertypical TECs. Wnt4 regulates the thymus master regulator Foxn1, which is required for embryonic TEC development (*58*), early postnatal mTEC expansion (*57*), and adult thymus maintenance and function (*64*).

In contrast, *Ly6d*^+^ intertypical TECs up-regulated the genes for fibroblast growth factor receptor 3 (*Fgfr3*) and 4 (*Fgfr4*) that bind fibroblast growth factors (Fgf) 8/9 (*65*) and 1 (*66*), respectively (Fig. 4F). Fgf signaling is required for thymus organogenesis (*67*–*69*) and has been implicated in TEC maintenance (*70*). Moreover, keratinocyte growth factor (KGF; also known as FGF7), along with Fgf10, binds to Fgfr2IIIb and induces transient thymus rejuvenation (*70*–*73*). *Fgfr2* is expressed broadly across intertypical TEC, cTEC, and immature mTEC subtypes (Fig. 4F), suggesting that KGF treatment acts on multiple TEC subtypes. Additionally, we observed that insulin-like growth factor signaling components are more highly expressed in *Ly6d*^+^ intertypical TECs, including potential transactivation between *Igf1r* and *Itga6* (Fig. 4, E and F) (*74*). *Itga6* is a marker of previously identified TEPC (*21*), supporting the notion that *Ly6d*^+^ intertypical TECs represent the progenitors previously described. Together, our analysis of the paracrine and autocrine signaling landscape across perinatal TEC subtypes highlights candidate signaling pathways that might separate the biological fates of *Ly6d*^+^ versus *Ly6d*^−^ intertypical TECs.

### *Ly6d*^−^ intertypical TECs are more affected by aging than *Ly6d*^+^ intertypical TECs

**Fig. 5.**
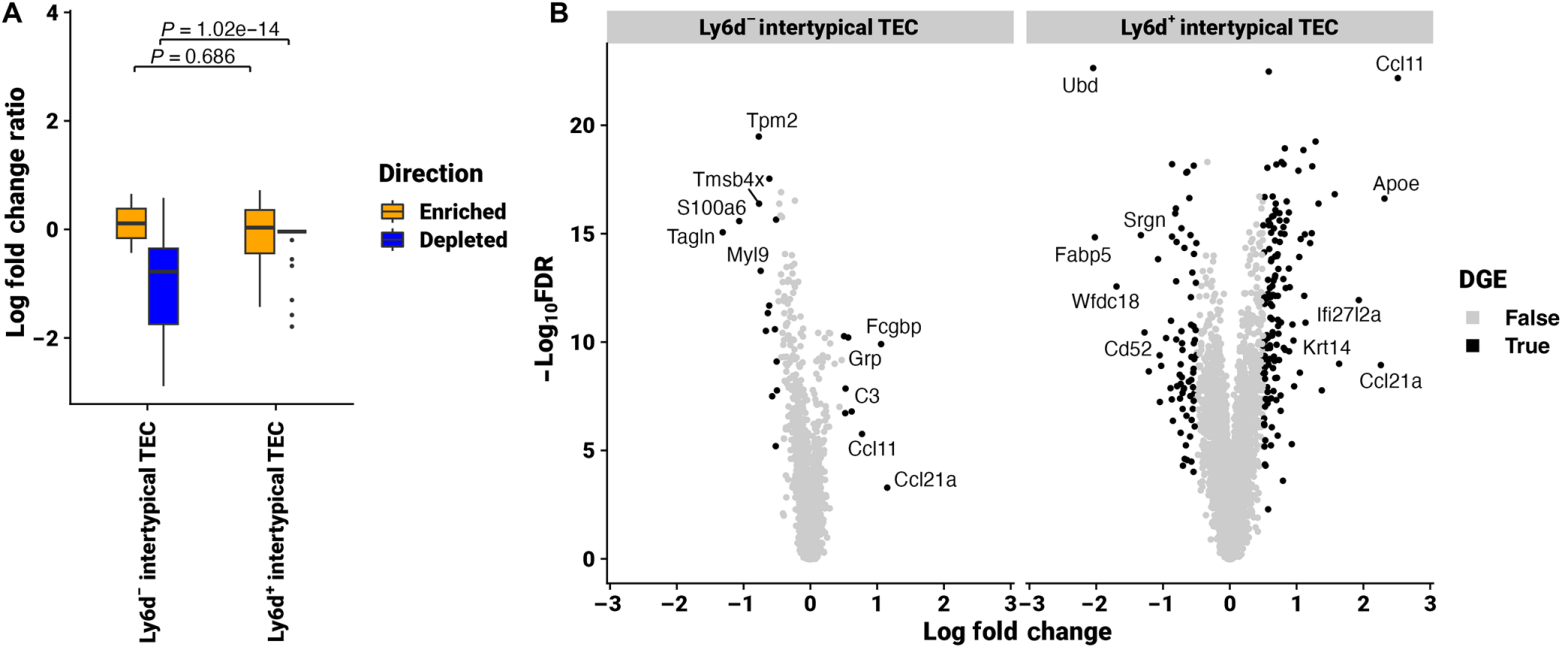
The impact of aging on *Ly6d*^+^ and *Ly6d*^−^ intertypical TECs. (**A**) DA log fold change ratio between ZsG^+^ and ZsG^−^ cells across the aging time course. Positive values denote a greater change in the ZsG^+^ and vice versa. Box plots are colored by whether the neighborhoods are concordantly enriched (orange), are depleted (blue), or are discordantly DA (orange). Boxes show the median, and box limits are the lower and upper quartiles. Whiskers extend to 1.5× the IQR with outliers beyond this range shown as individual points. (**B**) Volcano plots denoting DEGs across the mouse life course. Points are colored black with FDR < 0.1 or not DE (gray).

We have previously described an age-dependent accumulation of intertypical TECs (*12*). Therefore, we sought to understand the dynamics of our identified intertypical progenitor TECs across the mouse life course. First, we matched neighborhoods between our perinatal and aging datasets on the basis of the correlation of their transcriptomes (fig. S22A). We then performed DA testing to compare cells from younger to older mice separately on the ZsGreen^+^ and ZsGreen^−^ aging neighborhoods, which identified 1132 and 640 DA neighborhoods, respectively (10% FDR). This analysis showed that the abundance of both labeled and unlabeled cells is altered by aging (fig. S22, B and C). However, when focusing on specific TEC subtypes, we found that *Ly6d*^−^ intertypical TEC subsets were more depleted than the *Ly6d*^+^ intertypical TEC subsets in both the ZsGreen^+^ and ZsGreen^−^ fractions (Fig. 5A and fig. S22, D to F; *P* = 5.73 × 10^−28^, Mann-Whitney test), suggesting that *Ly6d*^−^ intertypical TECs might be more sensitive to the effects of aging. We additionally observed in older thymi a depletion along both the cTEC (immature cTEC, cTEC early, cTEC late, and cycling cTEC) and mTEC lineages (Sca1^+^ mTEC, Aire^+^ mTEC, and mTEC) as reported previously.

To understand the gene expression differences between younger and older intertypical TECs, we compared the transcriptomes of more versus less abundant *Ly6d*^+^ and *Ly6d*^−^ intertypical TECs separately, which identified 183 and 437 DEGs, respectively (Fig. 5B and fig. S23, 1% FDR). Coordinately up-regulated genes in more abundant neighborhoods of both intertypical TEC subtypes include *Nfib*, *Txnip*, and *Ackr4*; the latter sequesters *Ccl21a* (*75*, *76*), the canonical marker for intertypical TECs, whereas *Nfib* serves as an additional marker identifying *Ly6d*^−^ intertypical TEC (fig. S14B). Genes expressed in the less abundant neighborhoods were enriched for antigen presentation, including MHCII, and are generally up-regulated during TEC differentiation, in line with a restriction in lineage differentiation (*12*, *44*). GSEA (fig. S24) identified several cellular gene expression programs up-regulated by aging, including cytokine signaling [interleukin-6 (IL-6)/Janus kinase (JAK)/signal transducer and activator of transcription (STAT) signaling, interferon-α, interferon-γ, and TNF-α], response to mitogen and cell division (E2F targets, G_2_-M checkpoint, and Myc targets), and cell-cell communication [transforming growth factor–β (TGF-β) signaling and Wnt–β-catenin]. Based on Mohammed *et al.* (*52*), the disruption of both type I and type II interferon signaling in older thymi may contribute to the decline in TEC maintenance due to diminished hematopoietic cell input into the involuting thymus. Collectively, our results demonstrate the wide-ranging impact of aging on thymic epithelial precursors in keeping with inflammaging, replicative senescence, and remodeling of paracrine signaling. In sum, our analysis of TEPC, across the mouse life course, has identified the differential impact that aging has on *Ly6d*^+^ and *Ly6d*^−^ putative progenitors, providing molecular evidence for the disproportionate loss of mTEC during aging and thymic involution.

## DISCUSSION

**Fig. 6.**
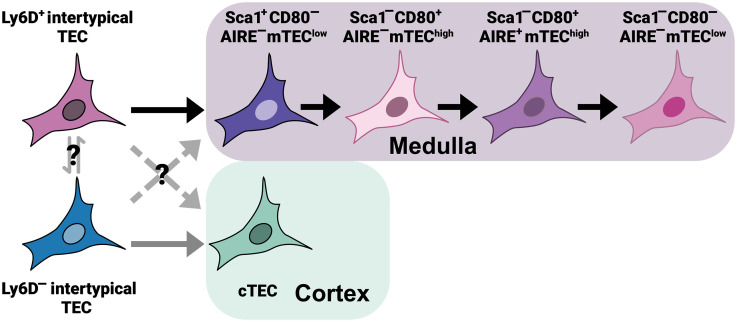
A schematic representation of the proposed lineage relationships between Ly6D^+^ and Ly6D^−^ intertypical TECs with major TEC lineages. Black arrows denote inferred progenitor-progeny relationships, and gray arrows denote unknown or yet-to-be tested relations.

TEC development and the search for thymic progenitor cell populations have attracted considerable attention in recent years due to its potential to unlock thymic rejuvenation. However, there is little consensus with regard to the identity and developmental potential of postnatal TEPC. Here, we have identified and quantified the contributions of β5t^+^ TEPC to postnatal TEC lineages in the mouse combining genetic lineage tracing with flow cytometry and single-cell transcriptomics. Our experiments reveal over the course of the first 9 weeks of life the disproportionate accumulation of lineage-trace labeled intertypical TECs compared to cTECs and mTECs (Figs. 1 and 2). These results suggest an early lineage bias switch in β5t^+^ TEPC from fetal cTECs to postnatal mTECs (Fig. 6). Supporting this conclusion, we resolve the identity of not one but two postnatal TEC progenitors, each distinguished by the expression of the surface marker Ly6d and their separate lineage biases (Fig. 3). Our transcriptome and spatial analyses of these cells extend to the impact of aging on each, in which we discover that *Ly6d*^−^ TEPCs are more affected than *Ly6d*^+^ TEPCs, leading to their greater depletion over 32 weeks of mouse life, a period that encompasses the rise and fall of thymic epithelium during involution (*12*).

We and others have previously shown that adult mTECs are derived from both fetal and newborn-derived TEC progenitors expressing the cTEC-specific protein β5t (*18*, *30*). Additionally, aging reduces the efficiency with which postnatal β5t^+^ TEPCs contribute to the maintenance of the thymus medulla (*18*, *30*). However, the phenotypic similarity between cTEC-like progenitor cells and other bona fide cTEC populations has limited the isolation and characterization of postnatal TEPCs that contribute to the medullary linage (*36*). Recently, we described intertypical TEC as a postnatal precursor to mature mTECs that arise from or contain β5t-expressing TEPCs (*12*). Intertypical TECs share marker genes with previously described TEPC populations, such as *Ccl21a*, Pdpn, and Sca1 (*19*, *20*, *29*, *44*). In this study, we used intracellular Ccl21 and surface Sca1 staining to flow cytometrically quantify the dynamics and contribution of β5t^+^ TEPC to intertypical TEC, cTEC, and mTEC subpopulations.

Detailed analysis of the labeling kinetics of cTECs, mTECs, and intertypical TECs showed an time-dependent accumulation of the ZsGreen label in the intertypical and mTEC compartments, whereas the proportion of ZsGreen^+^ cTECs decreased over the same period, indicating (i) a fast turnover of fetal-to-postnatal cTEC (*22*) and (ii) an increased contribution from β5t-low or negative progenitors postnatally (Fig. 1G). The constitutive expression of *Psmb11* during cTEC maturation and maintenance (*16*, *77*) prevents a definitive analysis of lineage relationships in the cortex using our transgenic system. However, using scRNA-seq of ZsGreen^+^ and ZsGreen^−^ sorted cells, we observed the same dynamics up to 10 days post–Dox treatment of 7-day-old pups, corroborating our findings.

Our model of *Psmb11*-dependent lineage tracing has the best discriminative power to discover progenitor-progeny relations for lineages that lack constitutive β5t expression yet are derived from *Psmb11* expressing precursors, such as mTECs and intertypical TECs. We observed a steady increase in ZsGreen^+^ mTECs across multiple developmental stages, which plateaus 4 weeks after Dox treatment. These results indicate that β5t^+^ TEPCs contribute equally to intermediate and mature mTEC subpopulations. We also observed a notable accumulation of ZsGreen signal in intertypical TECs, equivalent to a 50-fold increase over 8 weeks of lineage tracing (Fig. 2). More detailed flow cytometry analysis suggests that these accumulating cells are mTEC biased as the relative representation of ZsGreen^+^ cTEC-biased intertypical TECs remained constant (Fig. 2C). To explore these dynamics further, we applied scRNA-seq to our system, focused on the earliest time points with the greatest changes across TEC compartments.

Our single-cell transcriptome analysis identified two putative TEPC populations, delineated by the differential expression of *Ly6d*. Surface expression of Ly6D marks a number of progenitor populations in hematopoietic (plasmacytoid dendritic cells, lineage-restricted common lymphoid progenitors, and thymocytes) (*78*–*80*) and stromal cell types (prostate luminal cells) (*81*). Using bioinformatic analysis and DA testing, we found that *Ly6d*^−^ intertypical TECs accumulated in the ZsGreen^+^ fraction over time. In contrast, we observed no such bias for *Ly6d*^+^ intertypical TECs (Fig. 3). These analyses suggest that the latter subtype may precede a β5t^+^ state and that would place *Ly6d*^+^ intertypical TECs either as the earliest postnatal TEPC identified to date or as a parallel lineage that arises from a β5t^−^ TEPC. However, we observed a small fraction of ZsGreen^+^ *Ly6d*^+^ intertypical TECs, suggesting that some of these cells can be labeled. To explore this further, we found a small proportion of *Ly6d*^+^ intertypical TECs that express *Foxn1* and *Psmb11* transcripts, leading to the activation of our transgene after Dox treatment. Therefore, the balance of evidence indicates that *Ly6d*^+/−^ intertypical TECs contains the previously described β5t^+^ postnatal TEPC. Further lineage-tracing studies will be required to validate that these two TEC subtypes are able to give rise to mature postnatal cTECs and mTECs and to establish the relationship with each other. The marker genes identified in this work provide the basis to isolate and characterize lineage-biased TEPCs, and future efforts will need to be dedicated to validating the development potential of *Ly6d*^+^ and *Ly6d*^−^ progenitors.

We have previously described the acquisition of an inflammaging-like transcriptional signature in intertypical TECs, which we hypothesized might explain their reduced contribution to the mTEC compartment across the mouse life course (*12*). We further explored the impact of aging on our putative TEPC progenitors and found that *Ly6d*^−^ intertypical TECs are more strongly depleted across the mouse life course than *Ly6d*^+^ intertypical TECs. The down-regulated *Ly6d*^+/−^ intertypical TEC–expressed genes were indicative of TEC maturation, adding weight to previous observations that thymic involution is due to the restricted differentiation of progenitors into mature TECs (*12*, *18*, *30*, *44*). These results are important for strategies that seek to rejuvenate the aging thymus as they point to the dysfunction that requires correcting to restore the thymic epithelium and thus rejuvenate T cell development. Our results indicate these should inhibit inflammation (e.g., IL-6/JAK/STAT signaling), activate mitogen responses (e.g., Myc targets), and enhance cell-cell communication (e.g., TGF-β and Wnt–β-catenin signaling). Current experimental models to reinvigorate aged thymic cellularity include androgen depletion (*63*, *82*, *83*), Foxn1 overexpression (*84*, *85*), or growth factor treatment (*67*, *70*–*73*). The latter notably uses KGF (FGF7) that binds to Fgfr2-IIIb and acts as a mitogen that causes transient thymus growth and increased cellularity (*67*, *70*–*73*). Our data indicate that growth factor treatment may have wide-ranging effects on cTECs, immature mTECs, and intertypical TECs; whether the transient rejuvenation is caused by depleting the intertypical TEC is not clear and requires further investigation before KGF treatment can become a viable option for thymus renewal.

Our study makes extensive use of the 3xtg^β5t^ transgenic model for lineage tracing and scRNA-seq. This assumes that ZsGreen^+^ labeling is efficient and specific. Here, we show that efficient, yet differential, labeling is already achieved with low doses of Dox treatment, a feature that we use to our advantage to label maturing mTECs. However, the labeling of cells with no detectable *Psmb11* mRNA indicates that (i) scRNA-seq is limited at capturing very low expression levels and/or (ii) promoter accessibility without mRNA may be sufficient to permanently label cells. We observed labeled cells without *Psmb11* transcripts that correlated with the expression of *Foxn1*, the main regulator of *Psmb11* in TECs. Therefore, promoter accessibility may prime cells for the cTEC lineage without them irreversibly committing to it, restricting the 3xtg^β5t^ model.

Last, a true test of a bipotent TEPC is the ability of a single cell to give rise to both cTEC and mTEC lineages, given the right differentiation cues. Our experimental, single-cell, and spatial data all point to the presence of two unipotent or lineage-biased TEPCs, although a progenitor-progeny relationship between these TEC subtypes could not be firmly excluded. In sum, our detailed molecular and cellular characterization of Ly6d^+^ and Ly6d^−^ intertypical TECs now enables their further study to resolve their role in thymic maintenance across the life course.

## MATERIALS AND METHODS

### Mice

3xtg^b5t^ mice (β5t-rtTA::LC1-Cre::CAG-loxP-STOP-loxP-ZsGreen) mice have been previously described (*18*). Animals of both sexes were used. Mice were kept under specific pathogen–free conditions, with food and water ad libitum, at a temperature of 22° ± 2°C, 60 ± 15% humidity, and a 12-hour light cycle with light phase beginning at 6:00 a.m. with a 30-min sunrise and ending at 6:00 p.m. with a 30-min sunset. Both the caring for the mice and the experiments were performed in compliance with permissions and regulations of the Cantonal Veterinary Office of Basel-Stadt (license 2321).

### Dox treatment

Seven-day-old pups were treated with a single intraperitoneal injection if decreasing doses of Dox (0.3, 0.02, 0.004, or 0.0008 mg) diluted in 100 μl of sterile Hanks’ balanced salt solution (HBSS). Mice older than 4 weeks were treated with two intraperitoneal injections of Dox (2 mg, each) diluted in 200 μl of sterile HBSS. In the course of 24 hours in between Dox injections, the mice were also exposed to drinking water supplemented with Dox [2 mg/ml in sucrose (5% w/v)] (*18*).

### TEC isolation

Fatty and connective tissue was cleaned off the isolated thymi. Thymic lobes were cut into 0.1- to 0.2-cm cuboids using a scalpel and incubated either with Liberase and deoxyribonuclease I (DNase I; Roche Diagnostics; 200 and 30 μg/ml, respectively; for 45 min at 37°C) (*86*) or with papain (Sigma-Aldrich), Liberase, and DNase I (500, 200, and 30 μg/ml, respectively; for 30 min, at 37°C) (*87*) to obtain a single-cell suspension. Tissue digestion was mechanically aided by repeated pipetting of the cell suspensions at 15-min intervals. Last, cell suspensions were washed with Iscove’s modified Dulbecco’s medium (Life Technologies) containing 10% fetal calf serum (FCS) (HyClone, Thermo Fisher Scientific) and 2 mM EDTA (Invitrogen) to stop the enzymatic reaction and filtered through a strainer (70-μm pore size, Sefar Nitex) to remove debris. Cells were pelleted by centrifugation and resuspended in 0.2-μm pore-filtered phosphate-buffered saline (PBS) containing 2% (v/v) heat-inactivated FCS [designated fluorescence-activated cell sorting (FACS) buffer] for downstream analysis.

### Flow cytometry

Whole-thymus single-cell suspensions were typically stained with fluorochrome-conjugated antibodies against the desired surface epitopes for 30 min, at 4°C, in the dark. When required, cells were washed with PBS and stained with the appropriate Zombie fixable viability dye (BioLegend) for 30 min at 4°C before fixation. Fixation and permeabilization for intracellular staining were performed with either Cytofix/Cytoperm kit (BD Biosciences) or eBioscience transcription factor staining buffer set (catalog no. 00-5523-00, Thermo Fisher Scientific), according to the manufacturer’s guidelines. Specific antibodies and reagents, as well as the optimal conditions for their use (including dilutions), can be found in table S2.

### Immunofluorescence analysis

Frozen thymus tissue sections (7 μm) from 4- to 5-week-old C57BL/6 mice were dried overnight at room temperature, fixed in acetone for 10 min, rehydrated in PBS for 10 min, blocked in 5% goat serum in PBS for 20 min, and stained in blocking buffer using antibodies specific for CCL21 (polyclonal, LSBio), Ly6D (49-H4, BioLegend), Ly51 (6C3, BioLegend), and panCK (C-11, BioLegend). Streptavidin allophycocyanin (APC) (BioLegend) was used to detect biotinylated CCL21, and goat anti-mouse Alexa Fluor 750 secondary antibody (Invitrogen) was used to detect mouse panCK. Other antibodies were directly conjugated: Ly51FITC and Ly6DPE. Tissue sections were washed in PBS, stained with 4′,6-diamidino-2-phenylindole (DAPI; Merck), and mounted with Hydromount (National Diagnostics). Images were acquired using a Leica DMi8 microscope and analyzed using OMERO microscopy data management system at University of Basel.

### TEC sorting

Whole-thymus cell suspensions were generated by enzymatic digestion with papain, Liberase, and DNase I as described above. Cell suspensions were either enriched for EpCAM positivity or depleted of CD45^+^ cells before FACS using an automatic magnetic cell separator (AutoMACS Pro Separator, Miltenyi). Enriched cells were incubated for 30 min at 4°C in FACS buffer containing the required fluorescently labeled antibodies for the sort. After washing, cells were resuspended in FACS buffer and filtered through a 40-μm strainer into tubes containing DAPI (Invitrogen) for dead cell exclusion. Cells were sorted using a FACSAria II (BD Bioscience) into 1.5-ml Eppendorf tubes containing the most appropriate buffer for the desired downstream application.

### Droplet-based scRNA-seq

#### TEC processing and sorting

Whole-thymus single-cell suspensions from thymi isolated either 2 days or 10 days posttreatment with 0.004 mg of Dox were obtained by enzymatic digestion using papain, Liberase, and DNase I and enriched for TECs by EpCAM positivity, as described above. TotalSeq-A oligonucleotide-conjugated antibodies (BioLegend) were used to allow for barcoding and pooling of different TEC subpopulations, as well as to assess surface protein levels of different markers (table S3). Enriched cells were simultaneously stained with the required fluorescently labeled antibodies for the TEC sort as well as with the TotalSeq-A antibodies. TECs were subsequently sorted into four different subpopulations: ZsGreen^+^ Ly51^+^ UEA1^−^ cTEC, ZsGreen^−^ Ly51^+^ UEA1^−^ cTEC, ZsGreen^+^ Ly51^−^ UEA1^+^ mTEC, and ZsGreen^−^ Ly51^−^ UEA1^+^ mTEC. Postsorting, cell viability and concentration in each of the different samples were determined using a Nexcelom Bioscience Cellometer K2 Fluorescent Viability Cell Counter (Nexcelom Bioscience).

#### Library generation

Each well of a Chromium Single Cell B Chip (10x Genomics) was loaded with a total of 24,000 cells, pooled equally from each of the four sorted samples. The fact that the pooled samples had been barcoded with four different TotalSeq-A hashtag antibodies allowed to overload the 10× wells with 24,000 cells per well, aiming for a recovery of ∼10,000 single cells (∼40%) per well and enabling to overcome the increase in doublet rate by subsequently eliminating any cell barcode containing more than one single hashtag sequence from further analysis, as described in (*12*). The Chromium Single Cell 3′ Gel Beads-in-emulsion (GEM), Library and Gel Bead Kit (v3), and Chromium i7 Multiplex Kit (10x Genomics) were used for library preparation, according to the manufacturer’s instructions. In short, well number 1 on the Chromium Chip B was loaded with a mixture of the cell suspension with the GEM Retrotranscription Master Mix. Wells 2 and 3 were loaded with gel beads and partitioning oil, respectively. Nanoliter-scale GEMs containing the single cells to be analyzed were subsequently generated using the Chromium Controller (10x Genomics). Inside of each individual GEM occurs the simultaneous production of barcoded full-length cDNA from poly-adenylated mRNA as well as barcoded DNA from the cell surface protein-bound TotalSeq-A antibodies. Silane magnetic beads were used for the recovery and clean-up of the pooled fractions following the fragmentation of the GEMs, after which the recovered DNA was amplified and cDNA products, and Antibody-Derived Tags (ADT) and HTOs were separated by size selection. To optimize the amplicon size for the generation of the 3′ libraries, the full-length cDNA generated from polyadenylated mRNA was enzymatically fragmented and subjected to size selection. Subsequently, addition of P5, P7, a sample index, and TruSeq Read 2 (read 2 primer sequence) via end repair, A-tailing, adaptor ligation, and polymerase chain reaction (PCR) were performed to construct the libraries to be sequenced. In parallel, generation of the ADT and HTO libraries involved the addition of P5, P7, a sample index, and TruSeq Read 2 (read 2 primer sequence) by PCR according to the manufacturer’s instructions. Briefly, these are for ADT libraries 98°C for 2 min, 14 cycles of denaturing 98°C for 20 s, annealing 60°C for 30 s, and extension 72°C for 20 s, with a final extension at 72°C for 5 min. For HTO libraries, initial denaturing at 98°C for 2 min, then 13 cycles of denaturing at 98°C for 20 s, annealing at 64°C for 30 s, and extension at 72°C for 20 s with a final 72°C extension for 5 min. Amplicons for both ADT and HTO libraries were held at 4°C before downstream sequencing. The sequences of the oligonucleotides and primers used for this purpose can be found in table S4.

#### Library quality control, pooling, and sequencing

Capillary electrophoresis on a Fragment Analyzer (AATI) was used to assess the quality of the libraries generated before sequencing. The three sets of libraries generated were pooled as follows: 85% cDNA, 10% ADT, and 5% HTO, and the pooled libraries were sequenced using the NovaSeq 6000 S2 Reagent Kit (100 cycles) on an Illumina NovaSeq 6000 sequencing system (Illumina).

#### scRNA-seq quality control and processing

CITE-seq libraries were processed using Cellranger (v3.10) using mouse genome build GRCm38. Nonempty droplets were called using emptyDrops (v1.12.3) (*88*) on each multiplexed sample with 20,000 permutations, a lower unique molecular identifier (UMI) threshold of 100, and 1% FDR. Across all sequenced library samples, 101,255 nonempty droplets were defined (fig. S7). We defined low-quality single cells as those with <1000 UMIs and excessive mitochondrial RNA content (fig. S7). The latter was defined on the basis of departure from a normal distribution with mean set to the median mitochondrial RNA proportion of the sample and SD as twice the median absolute deviation. Excessive mitochondrial RNA content cells were then defined as those with a one-tailed FDR adjusted *P* < 0.05; 2627 cells were excluded from downstream analysis. HTO demultiplexing was performed as described in (*89*). Specifically, for each sequenced library, HTO UMI counts were scaled by HTO sequencing depth and used as input to *k*-means clusters, with *k* set to the number of expected clusters, i.e., the number of combinations of single and doublet HTO. For each HTO, we first assigned the candidate *k*-means cluster as the one with the highest normalized counts over cells. Excluding these cells and the top 5% of cells across other clusters, we then estimated a background distribution of counts by fitting a negative binomial with the fitdist function from the fitdistrplus package (v1.1-6) to the counts across the remaining cells. To assign cells to the HTO, we selected those with UMI count > 99th percentile. After repeating this for each HTO, cells were assigned to either singlet (a single HTO), multiplet (>1 HTO), or dropout (0 HTOs). For all downstream analyses, we excluded multiplet and dropout HTOs, leaving 64,500 high-quality cells.

#### Single-cell normalization, clustering, and visualization

Single-cell gene counts were combined across all samples and were rescaled using pooled estimate size factors before log + 1 transformation, as implemented in scran (v1.20.1) (*90*). HVGs were estimated by modeling the mean-variance relationship with modelGeneVar in the scran package, with a 10% FDR threshold: A total of 3327 HVGs were identified. Centered and scaled normalized gene counts for HVGs were used as input to randomized PCA, implemented in the R package irlba (v2.3.5). UMAP coordinates were computed using 50 principal components (PC) dimensions as input with 30 neighbors, min_dist = 0.2, and random initialized (seed = 42) as implemented in the uwot R package (v0.1.11). Single-cell clusters were calculated from a kNN graph computed using 30 PC dimensions and *k* = 21 using the Walktrap algorithm implemented in igraph (v1.2.9) (*91*, *92*).

#### DA and neighborhood analysis

Milo (v2.1.0) was used to build kNN graphs, construct refined neighborhoods (*k* = 50, props = 0.05) using the graph-only (*48*) method, and perform DA testing. Neighborhood counts were modeled using the Milo negative binomial generalized linear model (GLM), with a 10% FDR threshold, unless otherwise stated. Neighborhood clusters were defined by first constructing an adjacency matrix defined by an overlap threshold (overlap = 10), where an edge is drawn between pairs of neighborhoods sharing ≥ overlap numbers of cells. Louvain clustering was then performed on the corresponding graph using igraph (*87*). Neighborhood group marker genes were identified using the findNhoodGroupMarkers function in Milo with a 10% FDR threshold.

#### Cell-cell communication analysis

We inferred cell-cell communication between TEC subtypes using CellChat (v1.6.1) (*93*). We used the mouse CellChat DB to annotate expressed ligand and receptor genes across single cells. Gene expression data were averaged over TEC subtype labels using the trimean function, and clusters with fewer than 10 cells were excluded from analysis. Significantly communicating pathways were defined on the basis of a network out degree >10^−4^, which yielded 91 active signaling pathways in total. To estimate coordinated signaling modules, we used the cophenetic and silhouette indices to select a *k* value for subsequent NMF, with *k* = 4 used for both incoming and outgoing signaling module estimation. NMF factors were visualized as heatmaps using the ComplexHeatmap (v2.8.0) package (*94*).

#### Cross-study integration and neighborhood mapping

Gene counts data were downloaded for Baran-Gale *et al.* (*12*), Ragazzini *et al.* (*51*), and Wells *et al.* (*50*) and normalized gene expression data as described above. For Ragazzini *et al.* (*51*), we further used the multiBatchNorm function from the Bioconductor package batchelor (v1.8.1) to renormalize between datasets. HVGs were computed on each (Ragazzini *et al*., 8093; Wells *et al*., 2111; and Baran-Gale *et al*., 3614) and used as input for PCA using multiBatchPCA in the batchelor package. For Ragazzini *et al.* (*51*) and Wells *et al.* (*50*) data, we also integrated cosine-normalized principle components across studies using mutual nearest neighbors (*95*) (*k* = 50 and *k* = 21, respectively). kNN graphs were computed for each dataset (Ragazzini *et al*., *k* = 50; Wells *et al*., *k* = 21; and Baran-Gale *et al*., *k* = 50). We then computed refined neighborhoods using Milo (*47*, *48*) with the graph-only algorithm. To identify cross-mapping neighborhoods, we computed the Pearson correlation coefficient between neighborhood pairs, one each from our perinatal data and the corresponding study. We then identified the best matching neighborhood pairs as those with the highest Pearson correlation. Cross-mapping between TEC subtypes was visualized using ggalluvial (v0.12.3). For human to mouse cross-mapping, we first identified the one-to-one homologous genes from Ensembl, accessed through the Bioconductor package biomaRt (v2.48.3) (*96*).

#### Differential gene expression analysis

Single-cell gene counts were aggregated over groups, e.g., TEC subgroups and neighborhood clusters, for each replicate sample in each condition to generate pseudobulk counts. Differential expression testing was then performed by modeling pseudobulk gene expression counts using a negative binomial GLM, implemented in edgeR (v3.34.1) (*97*, *98*). DEGs were defined on the basis of statistical significance with a 10% false discovery threshold, unless otherwise stated. GSEA was performed on up- and down-regulated genes separately using the enrichR package (v3.0) (*99*). MSigDB Hallmark gene sets were used as input. Statistically significantly enriched/depleted gene sets were defined with a 10% FDR threshold. Differential gene expression (DGE) analysis results were visualized using ggplot (v3.3.5).

### Spatial transcriptomic profiling

A thymic lobe from an 8-week-old female C57BL/6 wild-type mouse was fixed in 10% neutral buffered formalin (Sigma-Aldrich) at 4°C for 24 hours and then embedded in paraffin. Sections at 5 μm thickness were loaded onto a Xenium slide (10x Genomics) and processed according to the manufacturer’s instructions for spatial transcriptomics using the Xenium Prime 5k Mouse Pan Tissue and Pathways Panel and the Cell Segmentation Staining kit (10x Genomics).

#### Spatial transcriptomics analysis

Cell types were initially annotated using label transfer from Steier *et al.* (*100*) before manually curating the final cell types to match the unsupervised clustering. The annotations were split into a preliminary cortical [DP (Q1), DP (Q2), DP (Sig.), DP (P), DN, cTEC, tc_stromal_mix(cor), CapFb, and Adipo] and a medullary compartment [mTEC, TlTEC, MedFb, Mature CD4, tc_stromal_mix(med), and SP4_resting/Treg-like] before progressing with the nearest-neighbor–based smoothening approaches (see below) to arrive at the final compartment definition.

#### Spatial transcriptome processing and analysis

The experiment was transformed into a Seurat object (v5) (*101*), filtered for cells with more than 50 and less than 600 unique features as well as cells with more than 100 and less than 900 total transcripts and subjected to Seurat’s SCTransform (*102*). Lifr and Ccl21a double-positive stromal cells (those that have nonzero raw counts for both these genes) were retained as intertypical TEC. To remove potential contamination from imperfect segmentation and transcript contamination from neighboring immune cells, we further filtered cells with nonzero expression for at least four of the following genes: *Ptprc*, *Cd8a*, *Cd8b1*, *Cd4*, *Cd3e*, *Cd3g*, *Rag1*, *Cd5*, *Cd69*, *Cd68*, *Sirpa*, *Lck*, *Tcf7*, *Gzma*, *Ighm*, *Ighd*, and *Itgax*.

Next, we took the intersection of the *Ly6d*^+^ and *Ly6d*^−^ intertypical TEC marker genes (table S5) with the genes covered by the Xenium 5k panel. One hundred eighty-nine Ly6d^+^ marker genes and 108 genes for Ly6d^−^ overlapped with the Xenium 5k panel. Module scores for both gene sets were computed per cell using the Seurat AddModuleScore function (*103*) and cell-wise subtracted from each other to create a joint score. The difference between module scores was normalized to the largest absolute number. Ly6d^+^ cells were classified based on a score of >0.1 and Ly6d^−^ intertypical as a score of <−0.1; all other cells were labeled as “unclassified.”

#### Thymic spatial compartment definition

A region definition for the thymus tissue section was developed exploiting the location of well-known cortical and medullary cell types (fig. S25). To obtain a global, cortex/medulla definition and overcome the mispositioning of few cortical cell types scattered in the medulla and vice versa, a nearest neighbor network [FNN package v1.1.4.1; (*104*)] was used to smoothen out inaccuracies in initial compartment allocation. This was done by querying the closest 11 cells to each other cell for their initial compartment annotation. Subsequently, the compartment annotation of the closest 501 cells was used to filter out small medullary islands. From this final cortex and medulla definition, we assigned cortical cells to the CMJ as those within 50 μm of a medullary cell. To compute the compartment area–scaled proportion of cells in each of the cortex, medulla, and CMJ, we counted the number of cells of each type (*Ly6d*^+^ and *Ly6d*^−^ intertypical TECs) and scaled these on the basis of the total cell area of each compartment. To calculate the total cell area for each histological compartment (cortex, CMJ, and medulla), the shortest distance from each cell centroid to the nearest neighbor cell was computed in each compartment separately and then averaged. This average shortest distance served as an estimate of the average diameter of each cell in each compartment. Next, we assumed hexagonal packing of cells and estimated the area using the following formula: Number of cells × $\frac{\sqrt{3}}{2}$ × ${\text{dist}}_{\text{avg}}^{2}$, where ${\text{dist}}_{\text{avg}}^{2}$ is the average squared physical distance between cell centroids.
